# The reproducibility of manual RV/LV ratio measurement on CT pulmonary angiography

**DOI:** 10.1259/bjro.20220041

**Published:** 2022-11-28

**Authors:** Sarah Lanham, Ahmed Maiter, Andrew J Swift, Krit Dwivedi, Samer Alabed, Oscar Evans, Michael J Sharkey, Suzanne Matthews, Christopher S Johns

**Affiliations:** Department of Clinical Radiology, Sheffield Teaching Hospitals NHS Foundation Trust, Sheffield, United Kingdom; Department of Clinical Radiology, Sheffield Teaching Hospitals NHS Foundation Trust, Sheffield, United Kingdom; Department of Infection, Immunity and Cardiovascular Disease, University of Sheffield, Sheffield, United Kingdom; Department of Clinical Radiology, Sheffield Teaching Hospitals NHS Foundation Trust, Sheffield, United Kingdom; Department of Infection, Immunity and Cardiovascular Disease, University of Sheffield, Sheffield, United Kingdom; INSIGNEO Institute for In Silico Medicine, University of Sheffield, Sheffield, United Kingdom; Department of Clinical Radiology, Sheffield Teaching Hospitals NHS Foundation Trust, Sheffield, United Kingdom; Department of Infection, Immunity and Cardiovascular Disease, University of Sheffield, Sheffield, United Kingdom; INSIGNEO Institute for In Silico Medicine, University of Sheffield, Sheffield, United Kingdom; Department of Clinical Radiology, Sheffield Teaching Hospitals NHS Foundation Trust, Sheffield, United Kingdom; Department of Infection, Immunity and Cardiovascular Disease, University of Sheffield, Sheffield, United Kingdom; INSIGNEO Institute for In Silico Medicine, University of Sheffield, Sheffield, United Kingdom; Department of Clinical Radiology, Sheffield Teaching Hospitals NHS Foundation Trust, Sheffield, United Kingdom; Department of Infection, Immunity and Cardiovascular Disease, University of Sheffield, Sheffield, United Kingdom; INSIGNEO Institute for In Silico Medicine, University of Sheffield, Sheffield, United Kingdom; Department of Clinical Radiology, Sheffield Teaching Hospitals NHS Foundation Trust, Sheffield, United Kingdom; Department of Clinical Radiology, Sheffield Teaching Hospitals NHS Foundation Trust, Sheffield, United Kingdom

## Abstract

**Objectives:**

Right ventricular (RV) dysfunction carries elevated risk in acute pulmonary embolism (PE). An increased ratio between the size of the right and left ventricles (RV/LV ratio) is a biomarker of RV dysfunction. This study evaluated the reproducibility of RV/LV ratio measurement on CT pulmonary angiography (CTPA).

**Methods:**

20 inpatient CTPA scans performed to assess for acute PE were retrospectively identified from a tertiary UK centre. Each scan was evaluated by 14 radiologists who provided a qualitative overall opinion on the presence of RV dysfunction and measured the RV/LV ratio. Using a threshold of 1.0, the RV/LV ratio measurements were classified as positive (≥1.0) or negative (<1.0) for RV dysfunction. Interobserver agreement was quantified using the Fleiss κ and intraclass correlation coefficient (ICC).

**Results:**

Qualitative opinion of RV dysfunction showed weak agreement (κ = 0.42, 95% CI 0.37–0.46). The mean RV/LV ratio measurement for all cases was 1.28 ± 0.68 with significant variation between reporters (*p* < 0.001). Although agreement for RV/LV measurement was good (ICC = 0.83, 95% CI 0.73–0.91), categorisation of RV dysfunction according to RV/LV ratio measurements showed weak agreement (κ = 0.46, 95% CI 0.41–0.50).

**Conclusion:**

Both qualitative opinion and quantitative manual RV/LV ratio measurement show poor agreement for identifying RV dysfunction on CTPA.

**Advances in knowledge:**

Caution should be exerted if using manual RV/LV ratio measurements to inform clinical risk stratification and management decisions.

## Introduction

Acute pulmonary embolism (PE) is common and can have high mortality rates if untreated.^1,2^ It represents a substantial burden on healthcare systems: in 2011, there were 28,000 hospital admissions and 250,000 bed days attributed to acute PE in the National Health Service (NHS).^3^ Consequently, there is great interest in ambulatory care models for patients with acute PE.^4^ These require accurate and reliable methods for risk stratification of patients. Several clinical scoring systems have been validated for risk stratification in acute PE, including the Pulmonary Embolism Severity Index and Bova score.^5–7^

The presence of right ventricular (RV) dysfunction is associated with high clinical risk and worse prognosis in acute PE, and is represented in the Bova score.^8–12^ A sufficiently large amount of embolus within the pulmonary vasculature increases the mean pulmonary artery pressure (MPAP) and thus afterload for the RV. Dysfunction occurs when RV output cannot be maintained by compensatory mechanisms. The resulting pressure overload causes RV dilatation which can be seen on imaging. RV dysfunction is exacerbated through multiple mechanisms, such as reduced left ventricular (LV) filling and output causing myocardial ischaemia. Dysfunction may progress to frank RV failure, the main cause of early death after acute PE.^12,13^

CT pulmonary angiography (CTPA) is the gold-standard investigation for the diagnosis of acute PE. Although neither a dedicated cardiac study nor routinely ECG-gated, CTPA may allow estimation of RV dysfunction. Accurate and reliable assessment of RV dysfunction on CTPA would enable diagnosis and risk stratification within the same investigation. An increased RV/LV ratio of >1 has been shown to correlate with the degree of RV dysfunction, and this quantitative measurement could aid objective risk stratification in acute PE.^12,13^ Few existing studies have attempted to assess the reproducibility of RV/LV ratio measurement and have been limited by the number and experience of reporters.^14,15^ This study aimed to assess the interobserver variability of manual RV/LV ratio measurement.

## Methods

### Study sample

Adult inpatient CTPA scans were retrospectively selected from a tertiary UK centre between 2017 and 2019. Cases were selected independently by two consultant thoracic radiologists. All scans had been performed routinely for patients referred for suspected acute PE. Ethical approval was not required for this retrospective study.

### Imaging procedures

#### CT protocol

Imaging was performed using a 20 detector-row CT system (Aquilion ONE/ViSION, Toshiba Medical Systems, Otawara, Japan) with the following standard protocol: 100 ml of intravenous contrast agent (Ultravist 300; Bayer Schering, Berlin, Germany) was administered at a rate of 5 ml s^−1^ and scanning was initiated at 3 to 14 s after attenuation in the pulmonary artery reached the threshold of 100 HU. Standard acquisition parameters were used: 100 mA with automated dose reduction, 120 kV, 1.0 pitch, 0.5 s rotation time, 1 mm slice thickness. A 400 × 400 mm field of view and 512 × 512 acquisition matrix were used and images were reconstructed with adaptive iterative dose reduction. The case images were anonymised and stored within the Picture Archiving and Communication System (PACS).

#### Image analysis

**Figure 1.**
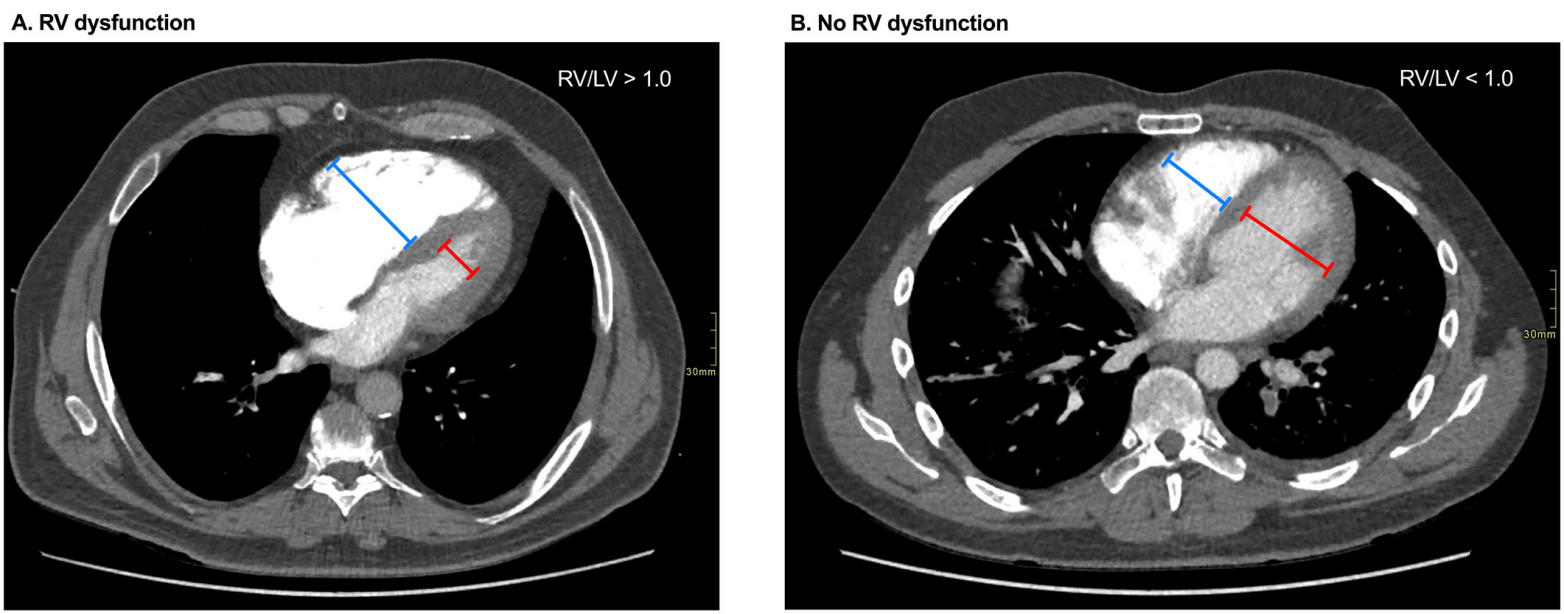
CTPA axial slices showing examples of RV/LV ratio measurements in two cases of bilateral PE. (**A**) increased RV/LV ratio >1.0 indicative of RV dysfunction; this case would be stratified as higher risk. (**B**) normal RV/LV ratio <1. *Blue measurement = RV cavity measurement, red measurement = LV cavity measurement*. CTPA, CT pulmonary angiography; LV, left ventricle; PE, pulmonary embolism; RV, right ventricle.

For each case, consultant and trainee radiologists within the centre were invited to participate in this study. They were requested to provide: (1) a qualitative opinion on the presence or absence of right ventricular dysfunction, and (2) manual quantitative measurement of RV and LV size and calculation of the RV/LV ratio, performed according to instructions (Figure 1). Images were reviewed on standard PACS workstations. RV and LV measurements were made on axial slices with the maximal internal diameters of the chambers (*i.e.* the widest point between the inner surfaces of the free wall and the interventricular septum). RV measurement was performed perpendicular to the long axis in the basal third of the cavity, with the tricuspid valve present on the image slice used. LV measurement was made with the mitral valve present on the image slice.

### Statistical analysis

Agreement of qualitative opinion on RV dysfunction was assessed using the Fleiss κ, with values interpreted in accordance with the following previously reported thresholds: 0.00–0.20 none, 0.21–0.39 minimal, 0.40–0.59 weak, 0.60–0.79 moderate, 0.80–0.90 strong, >0.90 almost perfect.^16^

The interobserver difference between RV/LV ratio measurements was assessed by two-way analysis of variance (ANOVA) with Tukey’s post-hoc test and their agreement was assessed using the intraclass correlation coefficient (ICC, two-way random effects model). ICC values were interpreted using the following previously reported thresholds: <0.50 poor, 0.50–0.75 good, 0.75–0.90 good and >0.90 excellent.^17^ Bland–Altman analysis was performed to assess the bias of the RV/LV ratio measurement for each reporter against the mean measurement for all other reporters combined. Using a threshold of 1.0, the RV/LV ratio measurements were classified as positive (≥1.0) or negative (<1.0) for RV dysfunction; the agreement of this classification was determined using κ.

One reporter (CSJ, chest radiologist with 10 years’ experience) repeated the RV/LV measurements for each case after a period of 6 months, with the ICC calculated to assess intraobserver agreement.

Analyses were performed using IBM SPSS (v. 28.0, IBM Corp., Armonk, NY). Results are reported as mean ± SD unless stated otherwise, with a significance threshold of 0.05. Graphs were produced using Prism 9 (GraphPad Software, San Diego, CA). All data were stored and analysed in accordance with the local data governance rules.

## Results

20 cases were included (60% female, median age 61 years). The clinical characteristics of the included cases are indicated in Supplementary Table 1. PE was present in 60% of CTPAs, with the majority bilateral (75%) and/or lobar (75%). A minority reported as showing evidence of RV dysfunction (42%) or associated lung infarction (33%). The other cases which did not demonstrate PE included evidence of pulmonary hypertension (PH, 20%), infective lung changes (10%) or no significant cardiorespiratory pathology (10%).

Responses were received from a total of 14 reporters: 3 cardiac consultant radiologists, 4 thoracic consultant radiologists, 2 gastrointestinal (GI) consultant radiologists and 5 trainee radiologists.

### Qualitative opinion on RV dysfunction

**Figure 2.**
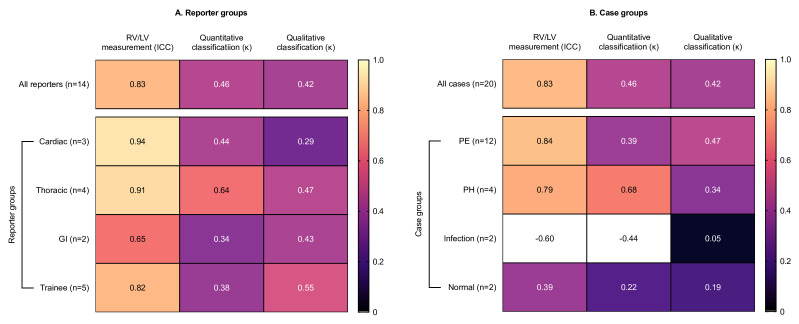
Interobserver agreement for reporter (**A**) and case (**B**) groups. Manual RV/LV ratio measurement showed good agreement (*left column*), but when used for classification of RV dysfunction, agreement was substantially worse (*middle column*). Classification based on overall qualitative scan opinion also showed weak agreement (*right column*). ICC and Fleiss κ values are interpreted in accordance with previously reported thresholds. ICC, intraclass correlation coefficient; LV, left ventricle; RV, right ventricle.

Qualitative opinion on the presence of RV dysfunction showed weak interobserver agreement (κ = 0.42, 95% CI 0.37 to 0.46; Figure 2). Agreement was higher between trainees (κ = 0.55, 95% CI 0.41 to 0.69) than between thoracic radiologists (κ = 0.47, 95% CI 0.29 to 0.65) or GI radiologists (κ = 0.43, 95% CI −0.01 to 0.87). Notably, agreement was minimal between cardiac radiologists (κ = 0.29, 95% CI 0.03 to 0.54). Agreement was weak for cases with PE (κ = 0.47, 95% CI 0.41 to 0.53), minimal for PH (κ = 0.34, 95% CI 0.24 to 0.45) and negligible for infection (κ = 0.05, 95% CI −0.93 to 0.20) or normal cases (κ = 0.19, 95% CI 0.05 to 0.34).

### RV/LV ratio measurements

**Figure 3.**
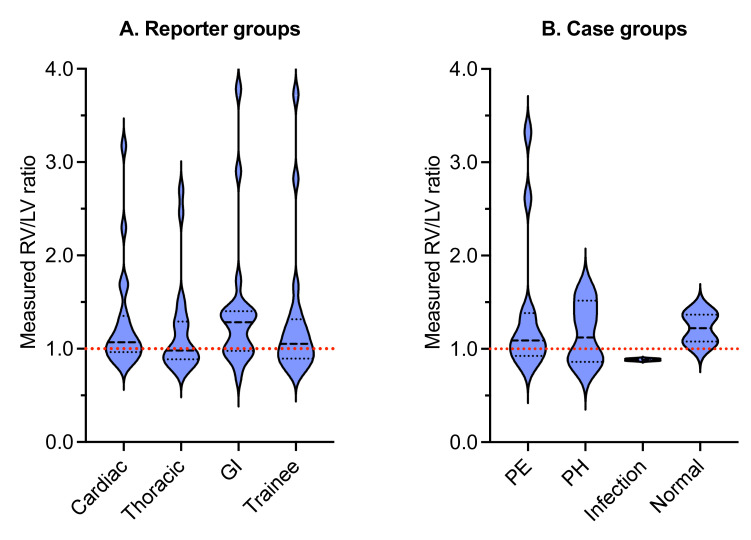
Violin plots of the RV/LV ratio measurements for cases. Quartile values (*dotted black lines*) and the threshold value of 1 are indicated (*dotted red line*) (**A**) measurements for each case for the different reporter groups. A significant difference was found between the thoracic and GI radiologist groups (*p* < 0.01). (**B**) measurements for each case, grouped by diagnosis on CTPA. No significant difference was found between the different groups (*p* = 0.35). CTPA, CT pulmonary angiography; GI, gastrointestinal; LV, left ventricle; PE, pulmonary embolism; PH, pulmonary hypertension; RV, right ventricle.

The mean RV/LV ratio measurement for all cases across all reporters was 1.28 ± 0.68, with a range of 0.59 to 6.00. The mean RV/LV ratios varied between all reporters (*p* < 0.001) and resulted in stratification of cases to different risk groups in 18 cases (90%). Figure 3 indicates the mean RV/LV ratio measurements for each case, grouped by case diagnosis and reporter group. Measurements were significantly higher for the GI radiologist group (1.40 ± 0.72) compared to the thoracic radiologist group (1.20 ± 0.52, *p* = 0.003); no other significant difference was found between reporter groups. No significant difference was found between the different case groups (*p* = 0.35). Bias values for RV/LV ratio measurements by each reporter are provided in Supplementary Table 2.

Interobserver agreement for RV/LV ratio measurement across all reporters was good (ICC = 0.83, 95% CI 0.73 to 0.91; Figure 2). This was higher for cardiac radiologists (ICC = 0.94, 95% CI 0.87 to 0.97) than for thoracic radiologists (ICC = 0.91, 95% CI 0.83 to 0.96), GI radiologists (ICC = 0.65, 95% CI 0.31 to 0.85) or trainees (ICC = 0.83, 95% CI 0.70 to 0.92). Agreement was good for PE (ICC = 0.84, 95% CI 0.71 to 0.94) and PH cases (ICC = 0.79, 95% CI 0.51 to 0.98), but poor for normal cases (ICC = 0.39, 95% CI 0.04 to 0.99). Disagreement was shown for cases of infection (ICC = −0.60, 95% CI −0.74 to 0.936). Intraobserver agreement for RV/LV ratio measurement was excellent (ICC = 0.95, 95% CI 0.88 to 0.98).

### Classification of cases by RV/LV ratio

In total, 12 cases (60%) were classified as positive for RV dysfunction according to the measured RV/LV ratios, using a threshold value of 1.0. Of these, three cases showed high RV/LV ratios of >1.50, suggesting severe RV dysfunction. Interobserver agreement of this classification was weak across all reporters (κ = 0.46, 95% CI 0.41 to 0.50, *p* < 0.001; Figure 2). This was moderate for thoracic radiologists (κ = 0.64, 95% CI 0.47 to 0.82), weak for cardiac radiologists (κ = 0.44, 95% CI 0.18 to 0.69) and minimal for GI radiologists (κ = 0.34, 95% CI −0.98 to 0.78) and trainees (κ = 0.38, 95% CI 0.25 to 0.52). Agreement was moderate for PH cases (κ = 0.68, 95% CI 0.57 to 0.78) but minimal for PE (κ = 0.39, 95% CI 0.34 to 0.45) and normal cases (κ = 0.22, 95% CI 0.07 to 0.36); disagreement was found for in cases with infection (κ = −0.44, 95% CI −1.90 to 0.1).

## Discussion

Identifying RV dysfunction is important for risk stratification of patients with acute PE. Manual measurement of the RV/LV ratio on CTPA may be performed to estimate the presence and degree of dysfunction at the time of diagnosis. This retrospective study assessed the reproducibility of manual RV/LV ratio measurements by reporters at a tertiary UK centre. 14 reporters evaluated 20 CTPA scans independently and recorded a qualitative opinion on RV dysfunction and a quantitative measurement of RV/LV ratio. Overall opinion on the presence or absence of RV dysfunction showed weak interobserver agreement. RV/LV ratio measurements differed significantly between reporters, with a wide range of values recorded for each case. When used to categorise cases for the presence or absence of RV dysfunction, measurement of RV/LV ratio also showed weak agreement, with stratification of cases to different risk groups in 90% of cases.

Qualitative opinion on the presence or absence of RV dysfunction showed weak agreement (κ = 0.42). Interestingly, agreement was lowest between cardiac radiologists, indicating the challenge of evaluating RV dysfunction on CTPA. While overall interobserver agreement for RV/LV ratio measurements was good (ICC = 0.83), agreement was weak when these values were used for classification of cases as either positive or negative for RV dysfunction (κ = 0.46 for all cases and κ = 0.39 for PE cases). In addition to the degree of agreement or disagreement, the κ value reflects the reliability of data measurements, and when κ values are <0.6, fewer than 35% of measurements may be reliable.^16^ This study suggests that manual RV/LV ratio measurement on axial slice orientation is poorly reproducible when used to determine the presence of RV dysfunction on CTPA. This is potentially problematic if this metric alone is used for clinical risk stratification, such as in ambulatory care pathways for acute PE. As with other quantitative imaging biomarkers, the reproducibility of measurement should be considered by both reporters and clinicians making patient management decisions.

Both qualitative opinion and classification by quantitative RV/LV ratio measurement showed poor agreement when identifying RV dysfunction. The former integrates multiple factors when assessing for the presence of RV function, such as thrombus burden, ventricular size, position of the interventricular septum and the presence of comorbid cardiorespiratory conditions.^18^ While quantitative biomarkers are appealing due to their perceived precision, they are still subject to disagreement and bias. Here, the weak agreement of RV/LV ratio measurement may be explained by several factors. Cardiac motion causes blur and slice-by-slice variation in the positions of cardiac structures on CTPA. Consequently, the slice chosen for measurement will also influence the RV/LV ratio value that is yielded. ECG gating of CTPA imaging may mitigate these effects and improve reproducibility, but is not routinely performed in the UK.^19,20^

We have demonstrated that manual measurement of the RV/LV ratio for the purpose of assessing RV dysfunction on axial views is poorly reproducible across a range of radiologists. These findings differ from those of Ende-Verhaar et al ^14^ and Kumamaru et al.^15^. Both studies demonstrated higher agreement of RV/LV ratio measurement (κ 0.83 in both), but assessed this between only four reporters (three trainee radiologists and one chest radiologist) and two reporters (a radiologist and a general practitioner) respectively. In comparison, this study included 14 reporters from a tertiary centre, across a range of experiences and including three cardiac subspecialty radiologists. Artificial intelligence (AI) methods may improve the reproducibility and reliability of this metric through automated measurement.^20,21^ AI approaches could enable more consistent risk stratification of patients according to biomarkers such as the RV/LV ratio and potentially integrate other radiological features to determine risk in acute PE.

We acknowledge the following limitations in this study. Only 20 cases from a single centre were assessed and a larger multicentre patient cohort may aid verification of these findings. Additionally, the non-blinded retrospective selection of cases carries the risk of selection bias. However, these factors may be outweighed by the number and variety of included reporters. We recommend that future studies implement blinding and randomisation in case selection to mitigate the risk of bias. As corresponding MPAP measurements for each case were unavailable, meaningful assessment of the diagnostic accuracy of RV/LV ratio measurement could not be performed. Future studies could attempt to validate our findings by comparing RV/LV ratio measurements on imaging with other measures of RV dysfunction, such as echocardiography or direct pulmonary artery pressure measurements from right heart catheterisation.

## Conclusion

Manual measurement of the RV/LV ratio on axial slices has poor overall reproducibility, which should be considered when interpreting CTPA scans in acute PE. Caution should be exercised if using manual RV/LV ratio measurements to inform clinical risk stratification and management decisions.

## Supplementary Material

bjro.20220041.suppl-01
