# Supplementary material for: The reproducibility of manual RV/LV ratio measurement on CT pulmonary angiography
Source: BJR Open. 2022 Nov 28;4(1):20220041. doi: 10.1259/bjro.20220041 (PMC10941330; doi:10.1259/bjro.20220041)
Supplement: bjro.20220041.suppl-01 [file bjro.20220041.suppl-01.docx]

**Supplementary Table 1**. Clinical characteristics of included cases and reported findings on CTPA.

| **Age (y)** | **Sex** | **CTPA findings** | | | |
| --- | --- | --- | --- | --- | --- |
|  |  | **Diagnosis** | **Features** | **Reported**  **RV dysfunction** | **Other findings** |
| 66 | F | PE | Bilateral lobar and segmental | Yes |  |
| 61 | F |  | Bilateral lobar | No | Post-radiotherapy lung changes |
| 53 | F |  | Bilateral central | No | Fluid overload |
| 38 | M |  | Unilateral lobar | No |  |
| 58 | M |  | Bilateral segmental | No |  |
| 61 | M |  | Bilateral lobar and segmental | Yes | Lung infarction |
| 61 | M |  | Unilateral lobar and segmental | No | Lung infarction |
| 57 | F |  | Bilateral lobar and segmental | Yes |  |
| 67 | M |  | Bilateral central and lobar | Yes | Lung infarction |
| 75 | F |  | Bilateral lobar and subsegmental | No |  |
| 80 | F |  | Bilateral lobar and segmental | Yes | Lung infarction  Infective lung changes |
| 52 | M |  | Unilateral segmental | No |  |
| 70 | F | PH | | Yes |  |
| 68 | F |  |  | Yes |  |
| 46 | F |  |  | Yes |  |
| 18 | M |  |  | No |  |
| 50 | F | Infection | | No |  |
| 56 | F |  |  | No |  |
| 82 | F | Normal | | No |  |
| 89 | M |  |  | No |  |

**Supplementary Table 2.** RV/LV ratio measurement bias for each reporter derived from Bland-Altman analysis. Bias is expressed as the % difference between the measured RV/LV ratio for each reporter compared to mean RV/LV ratio measurements of all other reporters.

| **Reporter** | **Role** | **Bias (%)** | **SD** | **95% CI Limits** | |
| --- | --- | --- | --- | --- | --- |
| A | Cardiac radiologist | -8.55 | 9.12 | -26.42 | 9.32 |
| B | Cardiac radiologist | 8.37 | 14.11 | -19.30 | 36.03 |
| C | Cardiac radiologist | 2.54 | 16.64 | -30.08 | 35.15 |
| D | Thoracic radiologist | -7.07 | 12.37 | -31.32 | 17.18 |
| E | Thoracic radiologist | -5.70 | 13.94 | -33.03 | 21.63 |
| F | Thoracic radiologist | -5.78 | 15.10 | -35.38 | 23.83 |
| G | Thoracic radiologist | -6.58 | 14.09 | -34.21 | 21.04 |
| H | GI radiologist | 12.27 | 18.04 | -23.09 | 47.63 |
| I | GI radiologist | 3.88 | 19.25 | -33.85 | 41.61 |
| J | Trainee radiologist | -2.25 | 14.47 | -30.62 | 26.12 |
| K | Trainee radiologist | 0.04 | 11.24 | -21.99 | 22.06 |
| L | Trainee radiologist | -9.36 | 17.14 | -42.96 | 24.24 |
| M | Trainee radiologist | -16.74 | 18.43 | -52.86 | 19.39 |
| N | Trainee radiologist | 16.06 | 20.85 | -24.81 | 56.93 |
